# Acute Effects of Inspiratory Loads and Interfaces on Breathing Pattern and Activity of Respiratory Muscles in Healthy Subjects

**DOI:** 10.3389/fphys.2019.00993

**Published:** 2019-08-02

**Authors:** Jéssica Danielle Medeiros da Fonsêca, Vanessa Regiane Resqueti, Kadja Benício, Guilherme Fregonezi, Andrea Aliverti

**Affiliations:** ^1^PneumoCardioVascular Lab/HUOL, Hospital Universitário Onofre Lopes, Empresa Brasileira de Serviços Hospitalares and Departamento de Fisioterapia Universidade Federal do Rio Grande do Norte, Natal, Brazil; ^2^Laboratório de Inovação Tecnológica em Reabilitação, Departamento de Fisioterapia, Universidade Federal do Rio Grande do Norte, Natal, Brazil; ^3^Dipartimento di Elettronica, Informazione e Bioingegneria, Politecnico di Milano, Milan, Italy

**Keywords:** respiratory muscles, healthy subjects, electromyography, plethysmography, physiology

## Abstract

**Objectives:**

The aim of this study was to evaluate the acute effects of different inspiratory loads and different interfaces on the breathing pattern and activity of the respiratory muscles.

**Methods:**

Twenty healthy adults were recruited and assigned to two groups (20 and 40% of the Maximal Inspiratory Pressure) by way of randomized crossover allocation. Subjects were evaluated during quiet breathing, breathing against inspiratory load, and recovery. The measurements were repeated using two different interfaces (nasal and oral). Chest wall volumes and respiratory muscle activity were assessed with optoelectronic plethysmography and surface electromyography, respectively.

**Results:**

During the application of inspiratory load, significant changes were observed in the respiratory rate (*p* < 0.04), inspiratory time (*p* < 0.02), minute ventilation (*p* < 0.04), tidal volume (*p* < 0.01), end-inspiratory volume (*p* < 0.04), end-expiratory volume (*p* < 0.03), and in the activity of the scalene, sternocleiomastoid, and parasternal portion of the intercostal muscles (RMS values, *p* < 0.01) when compared to quiet breathing, regardless of the load level or the interface applied. Inspiratory load application yielded significant differences between using nasal and oral interfaces with an increase in the tidal volume (*p* < 0.01), end-inspiratory volume (*p* < 0.01), and electrical activity of the scalene and sternocleiomastoid muscles (*p* < 0.01) seen with using the nasal interface.

**Conclusion:**

The addition of an inspiratory load has a significant effect on the breathing pattern and respiratory muscle electrical activity, and the effects are greater when the nasal interface is applied.

## Introduction

The application of an inspiratory load is a method used for inspiratory muscle training (IMT) (Hostettler et al., 2011); it helps to increase endurance and respiratory muscle strength in order to improve lung function (Sasaki, 2007), functional capacity (Shakouri et al., 2015), and efficacy in bronchial airway clearance (Oliveira et al., 2009). Three IMT techniques reported in literature are more commonly used: voluntary isocapnic hyperpnea, flow resistive loading, and pressure threshold loading (McConnell and Romer, 2004).

In inspiratory flow resistance devices, subjects perform inspirations through an orifice of variable diameter; the smaller the orifice diameter, the greater the resistive inspiratory load. However, these devices depend on the inspiratory flow generated by the subject. The new generation of electronic devices have overcome this limitation, in that, they are based on an electronically controlled variable flow resistive load. It has been reported that its use produces higher training loads and better inspiratory function when compared to pressure threshold devices (Langer et al., 2015). However, few studies have evaluated the compensatory mechanisms employed by the respiratory system when breathing is done against electronically controlled variable flow resistive loads.

The act of breathing is a vital biological process that, in normal conditions, occurs through the nose, for the purpose of filtering, humidifying, and heating the inspired air. Warmed, humidified air minimizes airway constriction and the resultant increased airway resistance. In addition, it improves upper airway mucociliary function, which augments the elimination of secretions. Despite this, the respiratory resistance devices available in the market only use mouthpieces (oral airway). The use of a nasal interface, for training inspiratory muscles, could be more favorable physiologically and more viable for individuals who are unable to hold a mouthpiece, such as patients with facial trauma or neurological problems that cause weakness of the facial muscles. Held et al. (2008) performed a protocol of muscular training and nasal breathing in mouth breathing children (MBS) and reported improvements in respiratory muscle strength and nasal respiratory flow. This study indicates that applied inspiratory loads using a nasal interface could induce compensatory mechanisms in the activities of respiratory muscles, resulting in an improved breathing pattern. However, the mechanism that provides these advantages when using a nasal interface is not well-understood.

Therefore, considering the possible benefits of nasal breathing on the respiratory system, we hypothesized that the use of a nasal interface will improve breathing pattern and the variation of chest wall volumes, as well as the electrical activity of the respiratory muscles.

## Materials and Methods

### Type of Study and Subjects

This research was carried out at the PneumoCardioVascular Lab, in the Federal University of Rio Grande do Norte/Brazilian Company of Hospital Services (UFRN/EBSERH) in the city of Natal/RN. We included, in the study, young adults belonging to the male and female sexes, presenting normal values of pulmonary function, with ages ranging from 18 to 30 years and with Body Mass Indexes (BMIs) between 18 and 29.9 kg/m^2^. We excluded individuals that failed to perform the tests/protocol, as well as those who presented irregularities during data analysis or voluntarily requested their removal from the study.

This study was approved by the Hospital Research Ethics Committee (number 428.987). The participants signed the consent form, which was drafted according to the tenets of the Helsinki declaration related to research on human beings, including special attention to confidentiality, respect for the human person, beneficence, autonomy, and non-maleficence.

### Pulmonary Function

Spirometry was performed using the KoKo DigiDoser^®^ spirometer (Longmont, CO, United States). The evaluations were performed according to the criteria of acceptability and reproducibility of the American Thoracic Society/European Respiratory Society (ATS/ERS) (American Thoracic Society/European Respiratory Society, 2002) and the reference values were derived from the predicted values for Brazilian adults (Pereira et al., 2007).

Respiratory muscle strength was determined by measuring maximal inspiratory pressure (MIP), maximal expiratory pressure (MEP), and sniff nasal inspiratory pressure (SNIP) using a digital manovacuometer (NEPEB-LabCare/UFMG, Belo Horizonte, Brazil). The evaluations were performed according to American Thoracic Society/European Respiratory Society (2002) acceptability and reproducibility criteria. Reference values previously published by Neder et al. (1999) were used for MIP and MEP, whereas the reference values of Araújo et al. (2012) were used for the SNIP. For all pulmonary function variables, the absolute values and percentage-predicted values were used for analysis.

### Optoelectronic Plethysmography

The measurement of the volumes of the chest wall (CW) and its compartments: the pulmonary rib cage (R_*Cp*_), the abdominal rib cage (R_*Ca*_), and the abdomen (Ab) was performed by Optoelectronic Plethysmography (OEP, BTS^®^, Milan, Italy), in which volumes were obtained following an experimental model according to the Gauss theorem (Cala et al., 1996). Before each data acquisition, the equipment was calibrated at a frequency of 60 Hz, the maximal frequency to achieve a quality signal for detecting chest wall movement. Six cameras positioned around the subject (three in the anterior region and three in the posterior region) captured the movement variation of 89 reflective markers. They were fixed at specific points in the anterior, posterior, and lateral regions of the thorax, between the clavicles and the anterior superior iliac spine, distributed in seven horizontal lines and in five pre-defined vertical columns, beside the addition of extra points. This setting was used to improve accuracy in volume assessment and to anatomically define the three regions or compartments of the chest wall, in which the frontiers between R_*Cp*_ and R_*Ca*_ is at the level of the xiphoid appendix and between R_*Ca*_ and Ab, along the costal margin anteriorly, and at the lowest point of the costal inferior margin posteriorly (Aliverti and Pedotti, 2003).

From the OEP data, the following variables were analyzed for the CW and its compartments: tidal volume [tidal volume in chest wall (V_*T,CW*_), tidal volume in pulmonary rib cage (V_*T,RCp*_), tidal volume in abdominal rib cage (V_*T,RCa*_), and tidal volume in abdomen (V_*T,Ab*_)]; end-inspiratory volumes [end-inspiratory volume in chest wall (EIV_*CW*_), end-inspiratory volume in pulmonary rib cage (EIV_*RCp*_), end-inspiratory volume in abdominal rib cage (EIV_*RCa*_), and end-inspiratory volume in abdomen (EIV_*Ab*_)]; end-expiratory volumes [end-expiratory volume in chest wall (EEV_*CW*_), end-expiratory volume in pulmonary rib cage (EEV_*RCp*_), end-expiratory volume in abdominal rib cage (EEV_*RCa*_), and end-expiratory volume in abdomen (EEV_*Ab*_)]; respiratory rate (RR); inspiratory time (T_*I*_); expiratory time (T_*E*_); inspiratory flow (Flow_*I*_); expiratory flow (Flow_*E*_); minute volume (MV); and total time of respiratory cycle (T_*TOT*_).

### Surface Electromyography (sEMG)

The surface electromyography (sEMG) was performed following the recommendations of the International Society of Electrophysiology Kinesiology (ISEK) (Merletti et al., 1999). Myoelectric signals were recorded using the electromyographic TeleMyo DTS Desk Receiver^®^ (Noraxon USA Inc., Scottsdale, AZ, United States) and four wireless sensors Clinical DTS (Noraxon USA Inc., Scottsdale, AZ, United States) with a 20–500 Hz pass filter-band, 1000 gain, 16-bit resolution, and a common mode rejection rate greater than 120 dB. Bipolar double trace Ag/AgCl (Miotec, Porto Alegre, Brazil) passive surface self-adhesive electrodes were placed on these muscles: scalene (SCL) at a distance of 5 cm from the sternum-clavicular joint and 2 cm above this point (Cunha et al., 2005), sternocleiomastoid (SCM) in the lower third of the distance between the mastoid process and the sternum-clavicular joint (Falla et al., 2002), abdominal rectus (RA) at 4 cm from the umbilical scar, and in the parasternal portion of the intercostal muscle (IC) over the second intercostal space and 3 cm from the sternum (Maarsingh et al., 2000), and all placed on the right side of the body to minimize cardiac noise interference. Before placing the electrodes, the skin region was prepared using an abrasive gel to reduce impedance to capturing the electrical signal. The software used to capture, process, and store the signals was MR 3.2 (Noraxon, Inc., Scottsdale, AZ, United States). Raw data was analyzed by means of RMS (root mean square) and standardized from respiratory baseline values (Soderberg and Knutson, 2000).

### Inspiratory Load

The effect of inspiratory loads with different interfaces was evaluated with an electronic variable resistive load device (POWERBreathe^®^ KH5, International, Ltd., Warwickshire, United Kingdom). The POWERBreathe < KH5 is an electronically controlled, variable flow resistance device generally used for IMT, in which an absolute initial load is assigned and successively reduced depending on the inspiratory flow generated by the subject.

### Randomization and Study Design

**FIGURE 1:**
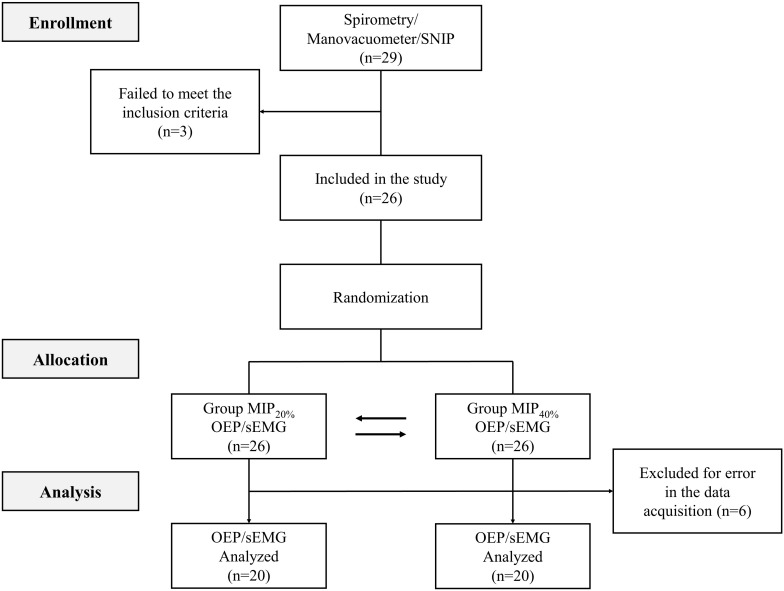
Study design.

The participants were categorized into two groups in randomized crossover design (MIP_20%_ and MIP_40%_). By way of a simple draw of an opaque envelope, they were grouped according to the initial resistive load applied (20 and 40% of MIP, respectively). Assessments were carried out in two stages: (1) clinical, spirometric, and respiratory muscle strength; and (2) chest wall volumes concomitant to the activity of respiratory muscles. During stage 2, the assessment consisted of three steps of 30 s each: (1) spontaneous quiet breathing (QB), (2) breathing against inspiratory load (Load), and (3) recovery (Rec) (Figure 1).

Due to the volume of the EMG signal data, with acquisitions at 1500 Hz and Optoelectronic Plethysmography at 60 Hz, the total duration for each step was limited to 30 s so the signals could be registered in a synchronized mode.

Two load intensities (20 and 40% of MIP) were applied and used with two different interfaces- oral and nasal, the order of which was designated by the same randomization process. The POWERBreathe was the resistive valve used for both interfaces. For the nasal interface, a patented device [PI 0164278 – INPI – Brazil], which consists of an orofacial mask associated with the resistive valve, was used. During its use, participants were asked to breath exclusively through the nose.

### Data Processing

A routine was developed in MatLab (MathWorks, Inc., Natick, MA, United States) to synchronize sEMG and OEP data. The resulting file was used to analyze the breathing pattern data, breath-to-breath data, on Diamov (a customized software designed and developed at the Politécnico di Milano, Italy). Furthermore, the file was also used to select the length of time and calculate RMS on MR 3.2 software.

The digital filters applied to EMG signals were electrocardiogram filter for cardiac electric signal removal, rectification, and smoothing filter for the elimination of non-reproducible signals (50 ms window).

### Sample Size and Statistical Analysis

Sample size was established considering the tidal volume as the main variable. Five subjects were evaluated using hypothetical one-way ANOVA during all three steps (QB, Load, and Recovery). A sample size ranging between 15 to 21 subjects for each group with a mean of 19 subjects was estimated using the following: an alpha error of 0.05 with bilateral distribution, and a test power of 80%, an effect size resulting from the ANOVA test described through the partial eta squared ($\eta_{p}^{2}$) values for the groups (MIP_20%_ with an oral interface (^$\eta_{p}^{2}$^ = 0.36), MIP_20%_ with a nasal interface ($\eta_{p}^{2}$ = 0.38), MIP_40%_ with an oral interface ($\eta_{p}^{2}$ = 0.42), and MIP_40%_ with a nasal interface ($\eta_{p}^{2}$ = 0.52).

Data normality was verified using the Shapiro–Wilk test. The Friedman test was used to analyze the steps of spontaneous quiet breathing, breathing against inspiratory load, and recovery, and in the case of a significant difference, Dunn’s *post hoc* test was applied. Comparisons between the oral and nasal interfaces, and that between 20 and 40% of the MIP loads were performed using the Wilcoxon test. For data analysis, the GraphPad Prism 6.0 program (GraphPad Software, San Diego, CA, United States) for Windows was used. The power (β) and effect size (ES) were estimated and are detailed in the results section of this study. For all statistical analyses, a level of significance, *p* < 0.05 with bilateral distribution was adopted.

The sample size calculation, β, and ES of the study were calculated using GPower software version 3.1.9.2 (University of Düsseldorf, Kiel, Germany).

## Results

Twenty-nine subjects were screened, of which three did not meet the inclusion criteria. Six subjects were excluded due to low quality of their electromyography signals and/or OEP volumes, resulting in a final sample of 20 subjects. The sample description and characterization referring to baseline anthropometric and pulmonary function data are shown in Table 1.

**TABLE 1:** Sample description.

Description	
Subjects (*n*)	20
Gender F/M	11/9
Age, yrs	24.4±2.72
BMI (kg/m^2^)	23.9±2.7
**Pulmonary function**	
FEV_1_ L	3.59±0.55
% predicted	95±9
FVC L	4.29±0.75
% predicted	95±10
FEV1/FVC	0.84±0.07
% predicted	100±8
MIP cmH_2_O	116.2±25.21
% predicted	104±24
MEP cmH_2_O	118±26.29
% predicted	98±10
SNIP cmH_2_O	104.8±19.4
% predicted	97±21

*Values are mean ± SD. F, female; M, male; yrs, years; BMI, Body Mass Index; kg, kilograms; m, meters; FEV_1_, Forced Expiratory Volume in the first second; L, liters; FVC, Forced Vital Capability; MIP, Maximum Inspiratory Pressure; cmH_2_O, centimeters of water; MEP, Maximum Expiratory Pressure; SNIP, Sniff Nasal Inspiratory Pressure.*

### Chest Wall and Compartmental Volumes

**FIGURE 2:**
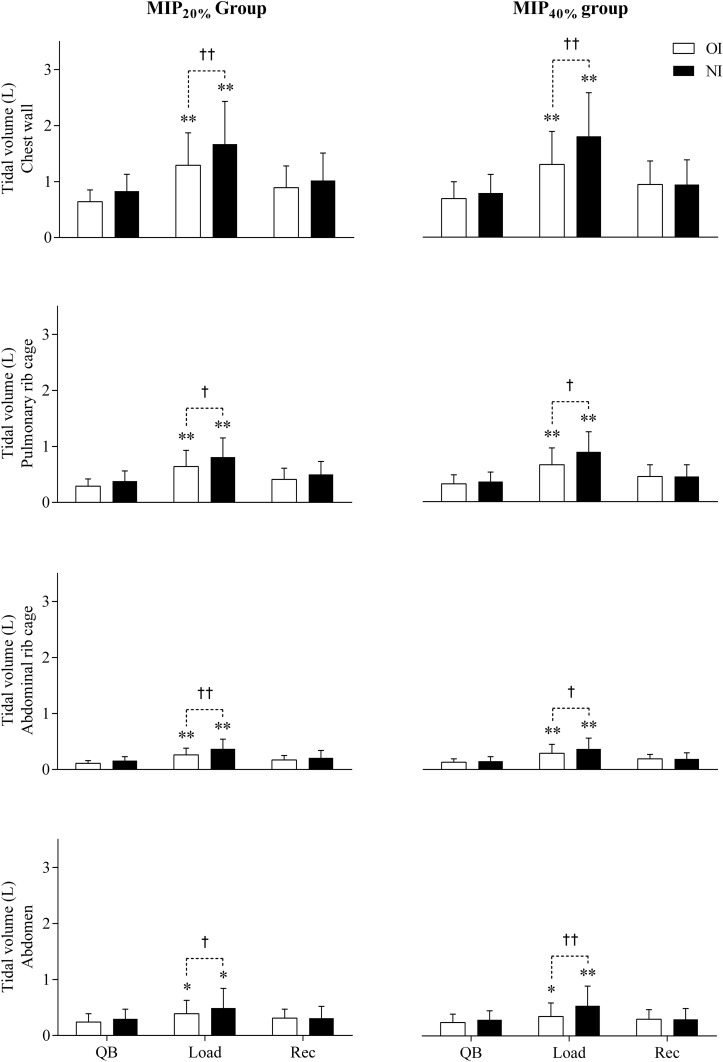
Variations of chest wall and its compartments tidal volume. QB, quiet spontaneous breathing; Load, breathing against inspiratory load; Rec, recovery; OI, oral interface; NI, nasal interface; L, liters. ^*^p < 0.05, ^∗∗^*p* < 0.01 for Friedman test with *post hoc* Dunne’s comparison with quiet spontaneous breathing. †*p* < 0.05, ††*p* < 0.01 for Wilcoxon test in the comparison between oral vs. nasal interface.

**FIGURE 3:**
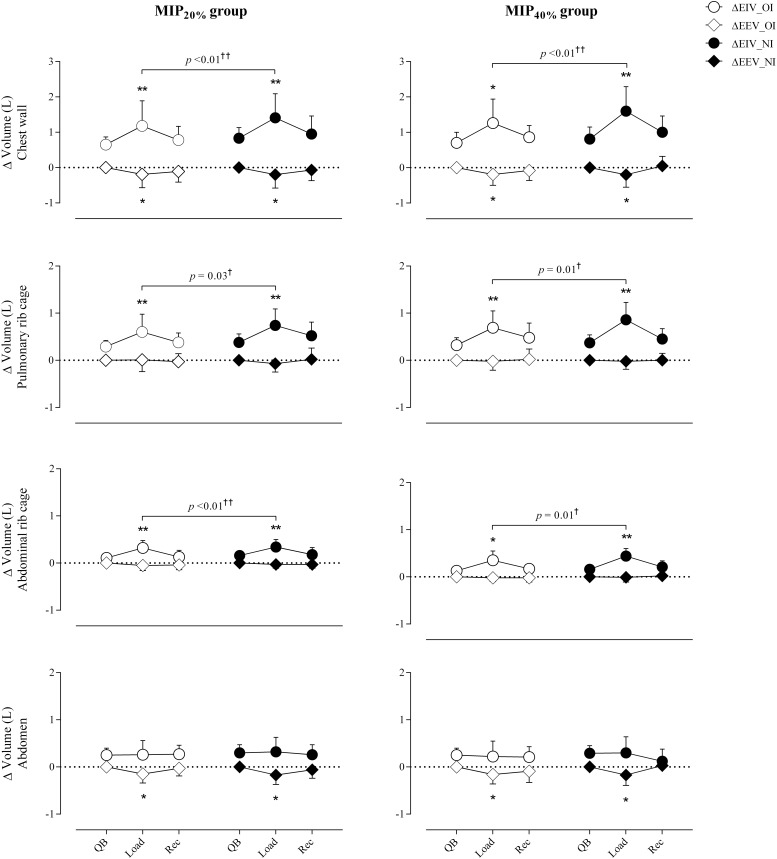
Variations of chest wall and its compartments operational volumes. QB: quiet spontaneous breathing; Load, breathing against inspiratory load; Rec, recovery; ΔEIV, end inspiratory volume variation; ΔEIV, end expiratory volume variation; OI, oral interface; NI, nasal interface. ^*^p < 0.05, ^∗∗^*p* < 0.01 for Friedman test with *post hoc* Dunne’s comparison with quiet spontaneous breathing. †*p* < 0.05, ††*p* < 0.01 for Wilcoxon test in the comparison between oral vs. nasal interface.

Figure 2 shows total chest wall and compartmental tidal volumes (V_*T,CW*_, V_*T,RCp*_, V_*T,RCa*_, and V_*T,Ab*_), while operational volume variations (end-inspiratory and end-expiratory total and compartmental volumes) are shown in Figure 3.

Regardless of the imposed load, significant increases in V_*T,CW*_ were observed during the Load step compared with the QB and Rec steps (*p* < 0.01). V_*T,CW*_ and compartmental volumes were higher when using the nasal interface (*p* < 0.05) compared with the volumes observed when the oral interface was used. The MIP_40%_ group presented an increase of 0.16 and 0.15 L with the oral and nasal interfaces, respectively, than the MIP_20%_ group. However, these values were not statistically significant.

Regarding the variations of operational volumes during breathing against inspiratory load, an increase in EIV_*CW*_ (MIP_20%_: oral and nasal interfaces, *p* < 0.01; MIP_40%_: oral interface, *p* = 0.04 and nasal interface, *p* < 0.01) and a reduction in EEV_*CW*_ (MIP_20%_: oral interface, *p* = 0.02 and nasal interface, *p* = 0.01; MIP_40%_: oral interface, *p* = 0.03 and nasal interface, *p* = 0.01) resulted.

During the Load step, there was a mean increase of 0.38 L in EIV_*RCp*_ (*p* < 0.01, regardless of the load intensity and interface used) and a mean increase of 0.22 L in EIV_*RCa*_ (MIP_20%_: oral and nasal interface, *p* < 0.01; MIP_40%_: oral interface, *p* = 0.02 and nasal interface, *p* < 0.01), when compared to the QB and Rec steps, resulting in a greater EIV_*CW*_. There was no significant variation in the abdomen. The EEV_*CW*_ reduction, in response to an increased ventilatory demand, was mostly related to the volume generated in the abdominal compartment (EEV_*Ab*_) (MIP_20%_: oral interface, *p* = 0.03 and nasal interface, *p* = 0.01; MIP_40%_: oral and nasal interface, *p* = 0.01). The abdominal compartment had a mean volume decrease of 0.16 L, whereas the rib cage maintained a constant volume.

Comparing the Load step when using the nasal and oral interfaces, EIV_*CW*_ was higher with loads imposed via the nasal airway (MIP_20%_ and MIP_40%_: *p* < 0.01), with no differences in relation to EEV_*CW*_.

As shown in Table 2, T_*TOT*_, T_*I*_, MV, Flow_*I*_, Flow_*E*,_ and RR varied during breathing against both inspiratory loads (MIP_20%_ and MIP_40%_) compared to QB, with only T_*TOT*_ not presenting significant variations with the MIP_40%_ load. T_*E*_ did not vary in any of the conditions. The comparison between different interfaces and load intensities did not show any statistically significant difference.

**TABLE 2:** Breathing pattern data obtained in quiet breathing, breathing against inspiratory resistance and recovery steps.

		MIP_20%_ group			*p-value*	MIP_40%_ group			*p-value*
		QB	Load	Rec		QB	Load	Rec	
T_*TOT*_ (s)	Oral interface	4.06 (3.3–5.4)	5.04 (3.93–7.56)	5.27 (4.17–6.13)	0.03^*^	4.39 (3.8–5.27)	6.34 (5.03–7.91)	4.48 (3.6–6.43)	*ns*
	Nasal interface	4.53 (3.76–5.79)	5.71 (4.52–8.59)	5.95 (3.5–6.81)	0.009^*^	4.84 (3.8–6.18)	6.59 (4.92–8.67)	3.92 (3–5.6)	*ns*
T_*I*_ (s)	Oral interface	1.76 (1.42–2.39)	2.46 (1.75–3.35)	1.8 (1.44–2.54)	0.006^*^	1.68 (1.44–2.08)	3.12 (2.29–4.09)	1.62 (1.27–2.39)	0.002^*^
	Nasal interface	1.80 (1.53–2.25)	2.74 (1.9–3.23)	1.99 (1.38–2.69)	0.008^*^	1.92 (1.55–2.7)	2.88 (2.1–3.99)	1.84 (1.48–2.6)	0.02^*^
T_*E*_ (s)	Oral interface	2.32 (1.79–2.59)	2.78 (2.07–3.78)	3.28 (2.57–3.95)	*ns*	2.73 (2.17–3.39)	3.08 (2.08–4.25)	2.8 (2.14–3.7)	*ns*
	Nasal interface	2.75 (2.15–3.12)	3.1 (2.33–4.74)	2.9 (2.06–4.15)	*ns*	2.67 (2.05–3.48)	3.28 (2.8–4.73)	2.96 (2.24–4.71)	*ns*
RR (bpm)	Oral interface	14.95 (11.7–18.3)	12.38 (8.05–15.8)	11.82 (10.53–15.27)	0.036^*^	13.97 (12.14–16.4)	9.68 (7.72–11.99)	14 (9.47–16.8)	0.02^*^
	Nasal interface	13.65 (10.5–16)	10.56 (7.23–13.4)	11.63 (9.23–17.28)	0.019^*^	13.25 (10.73–15.8)	9.18 (6.93–12.27)	15.6 (11.3–16.8)	0.002^*^
MV (L/min)	Oral interface	9.26 (6.79–12.17)	11.49 (9.49–6.6)	9.64 (7.08–13.32)	0.012^*^	8.53 (6.3–12.9)	11.01 (8.5–16.01)	11 (7.57–15.6)	0.015^*^
	Nasal interface	10.14 (8.64–13.2)	14.03 (11.1–24.7)	12.25 (9.4–4.45)	0.035^*^	10.12 (6.89–12.63)	14.67 (9.24–22.3)	12 (7.74–18.8)	0.004^*^
Flow_*I*_ (L/s)	Oral interface	0.32 (0.25–0.44)	0.45 (0.3–0.66)	0.46 (0.32–0.57)	0.001^*^	0.34 (0.26–0.49)	0.45 (0.4–0.62)	0.36 (0.26–0.59)	0.044^*^
	Nasal interface	0.42 (0.34–0.46)	0.5 (0.4–0.82)	0.49 (0.39–0.6)	0.011^*^	0.38 (0.28–0.46)	0.54 (0.36–0.93)	0.45 (0.3–0.56)	0,03^*^
Flow_*E*_ (L/s)	Oral interface	0.27 (0.19–0.37)	0.37 (0.30–0.55)	0.26 (0.17–0.37)	0.001^*^	0.22 (0.18–0.33)	0.4 (0.27–0.54)	0.32 (0.2–0.43)	0.02^*^
	Nasal interface	0.28 (0.22–0.39)	0.45 (0.33–0.65)	0.33 (0.24–0.43)	0.002^*^	0.31 (0.18–0.39)	0.5 (0.32–0.63)	0.25 (0.15–0.37)	0.001^*^

Data presented as median and interquartile range. RR, respiratory rate; T_*I*_, inspiratory time; T_*E*_, expiratory time; MV, minute ventilation; Flow_*I*_, inspiratory flow; Flow_*E*_, expiratory flow; s, seconds; L, liters; bpm, beats per minute; *ns*, not significant. ^*^p-values < 0.05 for Friedman test comparison between quiet breathing, breathing against inspiratory load and recovery steps.

### Electrical Activity of Respiratory Muscles

**FIGURE 4:**
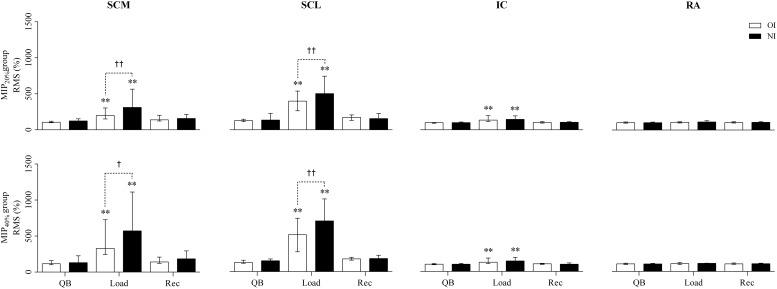
Percentage of respiratory muscle activation. QB, quiet breathing; Load, breathing against inspiratory load; Rec, recovery; SCM, sternocleidomastoid; SCL, scalene; IC, parasternal portion of the intercostal; RA, rectus abdominis. ^*^p < 0.05, ^∗∗^*p* < 0.01 for Friedman test with *post hoc* Dunne’s comparison with quiet breathing. †*p* < 0.05, ††*p* < 0.01 for Wilcoxon test in the comparison between oral vs. nasal interface.

Surface electromyography signals normalized by expressing RMS as percentage of resting conditions are shown in Figure 4. During breathing against both inspiratory loads, RMS of the SCM, SCL, and IC muscles increased (*p* < 0.01) in relation to the QB and Rec steps. The comparison between interfaces revealed significantly higher RMS values of the nasal interface for SCM (MIP_20%_, *p* < 0.01 and MIP_40%_, *p* = 0.01) and SCL (*p* < 0.01). sEMG signals were higher in MIP_40%_ than MIP_20%_ for SCM (*p* < 0.01), SCL (*p* < 0.01), and IC (*p* < 0.01). The RA muscle did not show any significant variations in its activity when different loads or interfaces were used.

### Power and Effect Size

Table 3 summarizes the effect size and power test for chest wall volumes and the sEMG RMS values of the SCM, SCL, and IC muscles during quiet breathing, load, and recovery steps. It also shows the comparison between the nasal and oral interfaces during the application of inspiratory load.

**TABLE 3:** Effect size and power test.

		MIP_20%_ group		MIP_40%_ group	
	
		Comparison between steps			
	
		Effect size Partial Eta $\eta_{p}^{2}$	Power	Effect size Partial Eta $\eta_{p}^{2}$	Power
V_*T*_	*Oral interface*	0.65	0.99	0.63	0.97
	*Nasal interface*	0.58	0.99	0.73	0.99
EIV	*Oral interface*	0.41	0.83	0.4	0.82
	*Nasal interface*	0.41	0.84	0.64	0.99
EEV	*Oral interface*	0.16	0.31	0.29	0.60
	*Nasal interface*	0.35	0.72	0.34	0.70
SCM	*Oral interface*	0.48	0.92	0.62	0.99
	*Nasal interface*	0.47	0.92	0.72	0.99
SCL	*Oral interface*	0.65	0.99	0.73	0.99
	*Nasal interface*	0.70	0.99	0.7	0.99
IC	*Oral interface*	0.44	0.89	0.56	0.98
	*Nasal interface*	0.59	0.99	0.43	0.86

		**Comparison between oral interface vs. nasal interface during load**			
		**Effect size Cohen’s *d***	**Power**	**Effect size Cohen’s *d***	**Power**

V_*T*_		0.86	0.94	0.84	0.93
EIV		0.62	0.73	0.66	0.78
EEV		0.17	0.11	0.04	0.10
SCM		0.70	0.83	0.6	0.70
SCL		0.73	0.86	0.74	0.86
IC		0.29	0.25	0.4	0.35

*Comparison of chest wall volumes and percentage of respiratory muscle activation intensity between quiet breathing, breathing against inspiratory load and recovery steps and between loading of nasal and oral interfaces. V_*T*_, tidal volume; EIV, end inspiratory volume; EIV, end expiratory volume; SCM, sternocleidomastoid; SCL, scalene; IC, parasternal portion of the intercostal; OI, oral interface; NI, nasal interface.*

## Discussion

We studied the acute effects of adding variable flow inspiratory loads using nasal and oral interfaces on the respiratory pattern variations and respiratory muscle activation. The addition of inspiratory loads resulted in: (1) increased tidal volume due to changes in the end-inspiratory volume and end-expiratory volume, the former occurring due to its increase in the R_*Cp*_ and R_*Ca*_ compartments, and the latter by its decrease in the abdomen compartment; (2) these changes were followed by increased electrical activity of the SCL, SCM and IC muscles; (3) these effects were more evident when using the nasal interface.

This study is a precursor to investigating the effects of inspiratory loads imposed on the nasal airway. We found that the nasal interface (nasal airway) promoted greater lung volume generation and inspiratory muscle activation when compared to the oral interface (oral airway). There are mechanical differences between the nasal and oral airways, depending on the region. The nasal airway has a higher resistance (RN = 0.68 cmH_2_O L^–1^ s^–1^ at 0.5 L s^–1^) and is relatively fixed, regulated by both the alar muscles and the nasal mucosa. In contrast, the oral airway presents a lower resistance (RO = 0.51 cmH_2_O L^–1^ s^–1^ at 0.5 L s^–1^); however, there is a high variability in resistance in response to mouth opening (Schiratzki, 2009; Strohl et al., 2012). A possible explanation for our results could be attributed to respiratory tract physiology, since the resistance resulting from nasal airway diameter is higher than that from the oral airway. This would increase the energy needed to generate inspiratory cycles, thus requiring a greater muscle recruitment to promote volume generation.

Thus, we can conclude that the effects of using the nasal interface were potentially better for the respiratory system. These results are aligned with those presented by Held et al. (2008). They demonstrated that a protocol of respiratory muscle training and nasal breathing performed in MBS children improved respiratory muscle strength and nasal inspiratory flow. These authors also reported the importance of nasal respiratory training for the reestablishment of lung volume and nostril elasticity in the studied subjects. Barbiero et al. (2007) observed improvements in forced vital capacity, Tiffeneau score, respiratory muscle strength, and daily habits in mouth breathers after therapy with re-expansive respiratory exercises associated with respiratory biofeedback. However, both studies used simplistic methodologies to evaluate the repercussions on the respiratory system and did not discuss the effects on breathing pattern or respiratory muscle activity.

The increase in chest wall (V_*T,CW*_) and compartmental (V_*T,RCP*_, V_*T,RCa*_, and V_*T,Ab*_) volumes was similar in both groups and although these volumes presented higher values in MIP_40%_ the change was not significant. Da Gama et al. (2013) analyzed the acute effects of increased inspiratory loads in healthy subjects using sEMG and OEP. Their results showed differences in volume generation depending on gender. It showed that for women, 20% of MIP is sufficient to generate significant changes, whereas for men, this change is only observed on using loads higher than 30%. Nobre et al. (2007) analyzed pulmonary ventilation using scintigraphy during inspiratory muscle endurance tests with 10, 20, and 30% of MIP in healthy women. Their results corroborate our results, suggesting that the use of inspiratory resistances greater than 20% of MIP was able to promote an increase in the pulmonary volume, and showed no significant differences between 20 and 30% of MIP as well.

The tidal volume is represented by the differences between end-inspiratory volume and end-expiratory volume generation (Wilkens et al., 2010). Alterations in respiratory drive occur in response to increased work demand resulting from inspiratory load imposition or physical exercise. In our subjects, both groups presented an increase in V_*T,CW*_ in response to an increase in the EIV in the rib cage compartments, due to the recruitment of the rib cage inspiratory muscles (SCM, SCL, and IC). By reducing the EEV, the abdomen compartment also contributes to the increase of the V_*T,CW*_. These results were previously described by Aliverti et al. (1997) in a study with healthy adults, which showed an increased EIV_*CW*_ and decreased EEV_*CW*_ in response to ventilation increased. These authors observed a reduction of 0.98 L in EEV_*CW*_ during exercise and identified the abdomen as the volume generator, which corroborates our study. Sanna et al. (1999) also reported changes in chest wall kinematics and respiratory muscles activity in healthy subjects during exercise, and attributed the ventilation increase to the recruitment of EIV_*Rcp*_ and EIV_*Rca*_, which are in agreement with our results. Both authors observed that in response to an increased ventilatory demand due to exercise, there is an increase in V_*T*_ of the rib cage associated with an increase in EIV, whereas the increase in V_*T,Ab*_ results only from a reduced EEV. They also suggested the importance of this arrangement in respiratory mechanics physiology.

The EEV_*Ab*_ reduction associated with the constant volume in the rib cage (R_*Cp*_ and R_*Ca*_) is a mechanism that supports the diaphragm muscle action. This increases the diaphragm pre-inspiratory length and prevents its excessive shortening during inspiration. In contrast, EIV increase associated with the rib cage volume, maintaining constant volume in the abdomen, favors the shortening of the chest wall inspiratory muscles during inspiration, preventing their excessive pre-inspiratory stretching (Aliverti et al., 1997). These authors also showed that EEV increase is a mechanism for optimizing ventilation regardless of exercise modality.

The electromyographic analysis improves the interpretation of our results. We found an increased activity in the SCM, SCL, and IC muscles during breathing against added load when compared to quiet breathing. This shows that the response of the rib cage muscles accompanied the increase in V_*T,CW*_ generation, being significantly higher in the SCL and SCM muscles when nasal interface was used, and with the 40% of MIP.

The chest wall muscles and the diaphragm need to act rhythmically and generate the necessary force needed to maintain ventilation (Han et al., 1993). Some studies evaluated muscle action by measuring the pressures generated by respiratory muscles during exercise (Aliverti et al., 1997; Sanna et al., 1999). They observed a progressive increase in the pressure generated by chest wall muscles during inspiration (Prcm). This was responsible for these muscles shortening, and consequently led to an increase in EIV, causing an expansion of the rib cage. The analysis of the abdominal muscle pressure (Pabm) showed an increase during expiration and reduction during inspiration. The former is related to the increase of EEV in the abdomen, whereas the latter associated with Prcm contributes to the expansion of R_*Ca*_ and an increase of EIV. The abdominal muscles also presented with a third function. Their relaxation during inspiration, associated with Prcm increase, allows the diaphragm to generate greater flow to meet the higher ventilatory demand.

Studies reporting the acute effects of inspiratory loads on the electrical activity of the respiratory muscles in healthy subjects were performed by Da Gama et al. (2013). In contrast to our results, they found a decrease in the activity of the SCM and diaphragm muscles in response to increased inspiratory load above 30 cmH_2_O. Their results were associated with a possible fatigue in these muscles. However, Nobre et al. (2007) reported similar results to our study, such that an increased activity in the lower rib cage muscles and the SCM were found, the latter not significant due to a higher inspiratory resistance. Walterspacher et al. (2018) evaluated the effect of IMT modalities on the activity of respiratory muscles, concluding that IMT promotes an increase of the SCM, IC, and diaphragm muscles activity. Using a pressure threshold device, Hawkes et al. (2007) reported the acute effects of submaximal inspiratory load on the inspiratory muscle strength of 12 healthy adult subjects. They found variations in the muscle recruitment pattern that was initially promoted by the diaphragm and later assisted by the intercostal muscle, which once activated, showed greater activity than the diaphragm.

Physiologically, the SCL and IC muscles have a primary inspiratory action and greater mechanical advantage (3.4 and 2.2 l^–1^, respectively) than the SCM (2.0 l^–1^). The secondary inspiratory function of the SCM, justifies its delayed recruitment. These values are representative of a mechanical advantage and were calculated by an indirect approach, based on the Maxwell reciprocity theorem. This theorem, when applied to the respiratory system, predicts that the potential change in the airway pressure produced by a particular muscle contracting alone against a closed airway is related to the mass of the muscle, the maximal active muscle tension per unit cross-sectional area, and the fractional change in muscle length per unit volume increase of the relaxed chest wall (De Troyer et al., 1998; Legrand et al., 2003). Another variable to be considered is the mass of the muscles, because, although the action of the IC is primary, its parasternal portion weighs only 3.2 g (De Troyer et al., 1998). Even though the IC is active from the beginning, the small mass could explain its reduced electrical activity during higher ventilatory demand when compared to other muscles. In contrast, the masses of the SCL and SCM muscles are approximately 33.2 and 62.2 g, respectively (Legrand et al., 2003). The fact that the SCM has almost twice as much mass as the SCL explains why they have similar muscular electrical activity despite the mechanical disadvantage of the SCM.

In summary, responding to inspiratory loads, the IC and SCL are initially recruited, but only the SCL responds by increasing its activation intensity according to the load. At loads greater than 20% of MIP, the SCM is recruited and has a similar intensity to the SCL due to its larger size. The SCM also increases its activity to match the load.

We did not find variations in the RA muscle activity in any of the comparisons made. Mesquita Montes et al. (2016) studied the effect of inspiratory and expiratory loads on the abdominal muscle activity in healthy subjects. They found results similar to ours, no variations on the RA muscle activity in response to inspiratory load, but an increased activity when using an expiratory load. Additionally, the authors described the behavior of the external oblique and transverse abdominus/internal oblique muscles. The latter reduced its activity when an inspiratory load was used, probably in order to minimize the effect of increased intra-abdominal pressure. This resulted in a greater recruitment of the diaphragm, thus revealing the contribution of the abdominal muscle during inspiration in response to increased ventilatory demand.

We did not analyze the activity of the external oblique, transverse abdominus, and internal oblique muscles. They are more sensitive to detecting abdominal muscle activity. This is a limitation to the present study. We used sEMG, which was not very effective in measuring the RA muscle activity, because of the thicker adipose layer covering. Another limitation to this study is the fact that the acute effects were assessed only for a limited period, which was necessary for the integrated analysis of the OEP and sEMG systems. However, the study presented favorable evidence on the use of inspiratory flow load and its application to upper airways, such as a better breathing pattern and greater muscle activation. An increase in lung volume mobilization in response the imposed load favorable to causing restrictive disorders was observed, without an increase in the end-expiratory volume and therefore, allowing its use also in obstructive disorders. In addition, we believe the nasal interface could be used as an alternative for treating patients with muscle weakness or facial deformities when it is not possible to use mouthpieces. Longitudinal studies must be performed in order to identify the long-term effects of inspiratory load application on ventilation and respiratory muscle strength.

## Conclusion

The results of our study indicate that applying inspiratory loads with a nasal interface is more effective in eliciting an increase on the inspiratory muscle activity, the chest wall and the compartmental volumes. As a perspective for future studies, we suggest evaluating the effects of IMT with a focus on the upper airways, in order to observe its repercussions on lung function.

## Ethics Statement

We declare that the above mentioned manuscript was approved by the Research Ethics Committee of the Federal University of Rio Grande do Norte under protocol 1.251.451/2015, according to the Declaration of Helsinki of 1975.

## Author Contributions

JdF: literature search, data collection, study design, analysis of data, and manuscript preparation. KB: analysis of data and manuscript preparation. VR: review of manuscript. GF: study design and review of manuscript. AA: literature search and review of manuscript.

## Conflict of Interest Statement

AA is a co-inventor of the optoelectronic plethysmography whose patent rights are held by his institution, the Politecnico di Milano. The remaining authors declare that the research was conducted in the absence of any commercial or financial relationships that could be construed as a potential conflict of interest.
